# A Deep Learning Framework with an Intermediate Layer Using the Swarm Intelligence Optimizer for Diagnosing Oral Squamous Cell Carcinoma

**DOI:** 10.3390/diagnostics13223461

**Published:** 2023-11-16

**Authors:** Bharanidharan Nagarajan, Sannasi Chakravarthy, Vinoth Kumar Venkatesan, Mahesh Thyluru Ramakrishna, Surbhi Bhatia Khan, Shakila Basheer, Eid Albalawi

**Affiliations:** 1 School of Computer Science Engineering and Information Systems (SCORE), Vellore Institute of Technology, Vellore 632014, India; bharanidharan.n@vit.ac.in (B.N.); drvinothkumar03@gmail.com (V.K.V.); 2 Department of ECE, Bannari Amman Institute of Technology, Sathyamangalam 638401, India; sannasi@bitsathy.ac.in; 3 Department of Computer Science and Engineering, Faculty of Engineering and Technology, JAIN (Deemed-to-Be University), Bangalore 562112, India; 4 Department of Data Science, School of Science Engineering and Environment, University of Salford, Manchester M5 4WT, UK; 5 Department of Engineering and Environment, University of Religions and Denominations, Qom 13357, Iran; 6 Department of Electrical and Computer Engineering, Lebanese American University, Byblos P.O. Box 13-5053, Lebanon; 7 Department of Information Systems, College of Computer and Information Science, Princess Nourah bint Abdulrahman University, Riyadh 11671, Saudi Arabia; sbbasheer@pnu.edu.sa; 8 Department of Computer Science, School of Computer Science and Information Technology, King Faisal University, Al-Ahsa 31982, Saudi Arabia; ealbalawi@kfu.edu.sa

**Keywords:** oral cancer, histopathologic images, CNN, deep learning framework, swarm intelligence, Gorilla Troops Optimizer

## Abstract

One of the most prevalent cancers is oral squamous cell carcinoma, and preventing mortality from this disease primarily depends on early detection. Clinicians will greatly benefit from automated diagnostic techniques that analyze a patient’s histopathology images to identify abnormal oral lesions. A deep learning framework was designed with an intermediate layer between feature extraction layers and classification layers for classifying the histopathological images into two categories, namely, normal and oral squamous cell carcinoma. The intermediate layer is constructed using the proposed swarm intelligence technique called the Modified Gorilla Troops Optimizer. While there are many optimization algorithms used in the literature for feature selection, weight updating, and optimal parameter identification in deep learning models, this work focuses on using optimization algorithms as an intermediate layer to convert extracted features into features that are better suited for classification. Three datasets comprising 2784 normal and 3632 oral squamous cell carcinoma subjects are considered in this work. Three popular CNN architectures, namely, InceptionV2, MobileNetV3, and EfficientNetB3, are investigated as feature extraction layers. Two fully connected Neural Network layers, batch normalization, and dropout are used as classification layers. With the best accuracy of 0.89 among the examined feature extraction models, MobileNetV3 exhibits good performance. This accuracy is increased to 0.95 when the suggested Modified Gorilla Troops Optimizer is used as an intermediary layer.

## 1. Introduction

Any neighbor tissue impairment due to uncontrolled cell growth and invasion is called cancer. Oral cancer is ranked as the sixth most prevailing cancer globally, and it falls under the broad category of head and neck cancer. Oral cancer results in malignant cell growth in the lips and various parts of the oral cavity. Worldwide, it is ranked as the fifteenth most common reason for death among various types of cancer. Out of one hundred thousand people, a minimum of four people are affected by this disease across the globe [1,2]. Approximately, seventy-seven new cases and fifty-two thousand deaths are registered every year in India, and one-fourth of the global oral cancer occurs in India [3]. In 2018, around 355,000 oral cancer cases occurred worldwide and resulted in 177,000 deaths. Estimates for the year 2020 include about 53,260 new cases added to the previous year’s cases, and the estimated death toll from this cancer in 2020 was about 10,750 deaths more than previous years [4,5].

The most common types of oral cancer include oral squamous cell carcinoma (OSCC), verrucous carcinoma, minor salivary gland carcinomas, lymphoma, and mucosal melanoma. Among them, OSCC is a predominant type of oral cancer which contributes around 84–97% of oral cancer cases [6]. The major risk factors that lead to the development of OSCC include tobacco usage, frequent chewing of betel quid, alcohol intake, oral infection, and genetic disorders [7]. Detection of OSCC at an early stage is very crucial to avoid deaths since the five-year survival rate of humans with early-stage OSCC is around 85%, while it is only around 40% with advanced stage [8,9]. Hence, early detection is the key to reducing the mortality rate and so there is a huge demand for diagnostic tools that identify OSCC at earlier stages of malignancy.

Apart from physical examination, major diagnostic tools used for the identification of oral cancer include techniques such as endoscope biopsy, liquid biopsy, the vital staining technique, ultrasound imaging, Magnetic Resonance Imaging (MRI), Computed Tomography (CT) imaging, Raman spectroscopy, gene/DNA array-based biomarker detection, enzyme assay-based biomarker detection, and histopathological examination [6]. Among these techniques, histopathologic examination is mainly preferred since it can be used to detect both malignant and benign tumors by identifying the changes in histopathological and molecular levels. Histological assays can be used to reveal the gradual growth of malignant cells in the oral cavity beginning from elementary dysplasia to tumors with a highly invasive nature. It helps analyze cell proliferation, growth of abnormalities, cytoplasmic-level and cellular-level atypia, changes at the surface of the epithelium, and deep tissue-level cytoarchitecture [10]. Usually, abnormalities at the microscopic and clinical levels arise only after abnormalities at the molecular and genetic levels. Histopathological examination is good at capturing these molecular-level changes and so is preferred for early detection [11].

Analysis of histopathological images with visual inspection is usually subjective and prone to errors sometimes. Particularly, the sensitivity and specificity measures will be very poor in the visual inspection of histopathological images when compared with computerized diagnostic tools. Sometimes, human inspection is biased so that many patients are identified as having OSCC even though they do not possess it, which makes the specificity measure low. In some other cases, the clinician may be biased to not detect a diseased person properly, which results in poor sensitivity. Computerized diagnostic tools will be very helpful to assist clinicians in the decision-making process to reduce such errors. Various machine learning (ML) techniques are used nowadays in a variety of fields. Particularly in the healthcare field, the implementation of ML algorithms is increasing day by day. Accuracy and robustness are the key concerns in such healthcare-related decision-making tools. Fortunately, nowadays, deep learning (DL) models are available for solving these issues. Deep learning is a sub-field in machine learning where Artificial Neural Network (ANN) models with many numbers of hidden layers are trained with a large set of training images and labels; labels of new unseen images are predicted using the trained model. The main advantage of deep learning is that it does not require hand-crafted feature engineering, which is required by traditional supervised classifiers such as Support Vector Machine (SVM), K-Nearest Neighbor (KNN), Decision Trees (DT), etc., where domain experts are required to identify the appropriate features and Region of Interest (RoI) [12,13].

Convolutional Neural Networks (CNNs) are a popular deep learning technique where convolution operation is involved in multiple ANN layers. Various CNN architectures have been developed, and they are very efficient in different image classification tasks [14,15]. Popular CNN architectures include ResNet, EffiecientNet, InceptionNet, MobileNet, etc. The main advantage of these architectures is their ability to work well on a classification task even if most of their weights are pre-trained on another classification task. This concept is known as transfer learning and works very well for two similar and unique classification tasks. The two main advantages of transfer learning are a reduction in training time and competence to work well on small datasets [16,17].

To improve the accuracy of such deep learning models, various techniques are used such as fine-tuning, feature selection, regularization, optimal parameter selection, optimization, etc. On the other hand, various population-based swarm intelligence (SI) optimization algorithms are widely used for optimal parameter identification, weight updating, and feature selection in deep learning models for enhancing accuracy. SI algorithms are meta-heuristic iterative algorithms that are usually inspired by the characteristics and nature of the swarm of animals. These algorithms are preferred in many applications, mainly due to their minimalism, derivation-free design, and ability to avoid local optima [18]. Some of the popular SI algorithms include Particle Swarm Optimization (PSO), Ant Colony Optimization, the Grey Wolf Optimizer, Dragonfly Optimization, Elephant Herding Optimization, the Gorilla Troops Optimizer (GTO), etc.

This work primarily focuses on classifying histopathological images into two categories: Normal and OSCC. Histopathological image features are extracted using pre-trained weights of transfer learning-based popular CNN models namely, InceptionV2, MobileNetV3, and EfficientNetB3. Then, the Modified Gorilla Troops Optimizer (MGTO) is used as an intermediate layer in between feature extraction and classification layers. Two fully connected ANN layers, batch normalization, and dropout are used as classification layers.

The key contributions of this research work are listed below:

1. The proposal of a novel deep learning framework that includes a swarm intelligence-based optimization algorithm as an intermediate layer in the deep learning model.
2. The development of MGTO with appropriate modifications that enhance classification accuracy.
3. A comparative analysis of popular deep learning models with and without the proposed intermediate layer in terms of various classification metrics and training times.

The remainder of this paper is organized as follows: Section 2 deals with related works, and Section 3 is related to the background of various transfer-learning models used and the original GTO. Section 4 deals with the methodology used in this research work, and Section 5 presents the implementation procedure for the proposed MGTO as an intermediate layer. Results are presented and discussed in Section 6. The last section summarizes the conclusion and future work.

## 2. Related Work

Various techniques based on machine learning and deep learning are proposed in the literature to diagnose oral cancer by analyzing medical images. Early publications related to oral cancer diagnosis mainly use feature extraction and traditional supervised classifiers [19,20,21,22]. For example, Krishnan et al. [23] considered features based on texture discrimination using higher order spectra, laws texture energy, and local binary pattern and fed these features to supervised classifiers such as DT, the Gaussian Mixture Model, KNN, the Sugeno Fuzzy Classifier, and the Radial Basis Probabilistic Neural Network. Similarly, Thomas et al. [24] proposed textural change detection using features extracted from digital images of oral lesions using a grey-level cooccurrence matrix and grey-level run length matrix. They used back a propagation-based ANN for classification. Particularly for OSCC diagnosis, Rahman et al. [25,26] proposed texture, shape, and color feature extraction from histopathological images and classification using DT, SVM, and Logistic Regression.

The usage of deep learning models in medical image analysis is increasing rapidly, particularly from the last decade onwards. Various deep learning models are developed and tested for oral cancer diagnosis that involve both binary and multi-class classification. In [27], the authors investigated a customized AlexNet model for detecting OSCC from histopathological images. In [28], the authors applied the DenseNet121 model to oral biopsy images to detect OSCC and found that it performs better than regions with the CNN (R-CNN) model. Other transfer learning models such as Inception-ResNet-V2 [29], Xception [30], and ResNet101 [31] were also investigated for diagnosing oral cancer from medical images. Apart from the above-mentioned works where popular CNN architectures are investigated, some works propose their own CNN model for detecting oral cancer. For example, Lin et al. [32] proposed the HRNet model for diagnosing malignant lesions in oral cavities and compared it with popular ResNet50 and DenseNet169 models. In [33], the authors developed a modified CNN model that performs well when compared with transfer learning-based models such as Resnet-50, VGG-16, VGG-19, and Alexnet. Similarly, Das et al. [31] proposed their own ten-layer CNN model that outperforms pre-trained CNN models in the diagnosis of OSCC from histopathological images. Other than CNN and its variants, capsule networks are also implemented in some works to identify oral malignancy. In [34], the authors tested performance capsule networks to identify OSCC from histopathological images.

Many optimization algorithms are used in many applications to enhance the classification performance and robustness of deep learning models, and some of them are outlined below. A hybrid optimization algorithm was developed that mixes PSO and Al-Biruni Earth Radius Optimization for optimizing the design parameters of CNNs and Deep Belief Networks in malignant oral lesion identification [35]. The segmentation of psoriasis skin images using Adaptive Golden Eagle Optimization was implemented for finding the ideal weight and bias parameters of CNNs [36]. The Artificial Bee Colony optimization algorithm was considered for finding the optimal hyper-parameters of a CNN that worked as a classifier for identifying plant species [37]. The optimal guidance-whale optimization algorithm was used to select features extracted from an AlexNet–ResNet50 model, and the selected features were supplied to bi-directional long short-term memory for Land Use Land Cover classification [38]. Modified Lion Optimization was implemented for selecting the optimal features in a transfer learning-based CNN classification model to build a multimodal biometric system [39]. In this manner, numerous optimization algorithms are incorporated for finding optimal hyper-parameters, training models, and feature selection in deep learning. Comparatively, only a few works are reported regarding the usage of optimization algorithms as a transformation technique. For example, the Crow search optimization algorithm is used was a transformation technique for improving the classification performance of weighted KNN in the severity classification of breast cancer [40].

From the above-related works, the following points can be summarized. Compared with hand-crafted feature extraction and traditional supervised classifiers, deep learning models perform well in the diagnosis of OSCC. But still, they lag in classification accuracy and robustness. To solve these two concerns, optimization algorithms are widely used in various applications for the improvisation of deep learning models in different ways. Hence, this work attempts to use the MGTO optimization algorithm as an intermediate layer between feature extraction and classification layers for enhancing the accuracy of OSCC diagnosis.

## 3. Background

### 3.1. CNN

CNN [41]-based deep learning models are widely used to classify images in a variety of applications, mainly due to their capability of recognizing an underlying pattern. Convolution operation at multiple layers acts as the foundation for CNN and generally, a typical CNN contains convolutional layers, pooling layers, and fully connected layers. The goal of convolution layers is to extract the image attributes such as contours, colors, etc. Pooling layers act as a dimensionality reduction layer, i.e., the layer that reduces the number of features. Max and average pooling layers are very popular when compared with others. The last stage is usually built using a fully connected layer called DenseNet, and it is responsible for classification [42].

### 3.2. InceptionV2

The inception [43] model is an altered version of a CNN in which inception blocks are included. These inception blocks refer to the processing of the same input with different filter sizes before combining them. InceptionV2 is an advanced variant of the original InceptionV1. When compared with Inception V1, two 3 × 3 convolution operations are performed in Inception V2 instead of one 5 × 5 convolution operation. In addition, the filter size *n* × *n* is factorized into 1 × *n* and *n* × 1 convolutions in Inception V2.

### 3.3. MobileNetV3

MobileNet [44] is a modified version of a CNN where batch normalization and ReLU activation functions are used instead of a single 3 × 3 convolution layer. In addition, one convolution operation is carried out for each color channel in MobileNet, while the flattening of color channels will happen in typical CNNs. Relatively, MobileNet architectures require minimal computational power and so are mainly preferred in mobile devices and embedded systems. Compared with MobileNetV1, bottlenecks with residuals are implemented in MobileNetV2, while layer removal and swish non-linearity are incorporated in MobileNetV3.

### 3.4. EfficientNetB3

Unlike typical CNNs, EfficientNet [45] uniformly scales all dimensions with a compound coefficient. A fixed set of scaling coefficients is used to uniformly scale the network depth, width, and resolution. The original EfficientNetB0 version is based on MobileNetV2 combined with squeeze and excitation blocks. EffientNetB3 is developed by scaling up the baseline network of previous versions.

### 3.5. Gorilla Troops Optimization

GTO is one of the iterative meta-heuristic optimization algorithms that was proposed in the year 2021 [46]. It is based on the social activities and characteristics of a gorilla troop. Usually, each such troop contains one adult male gorilla, called a silverback gorilla, a substantial number of adult female gorillas, and their children. The male gorilla leads the troop, and it is responsible for controlling the troop activities such as identification of sources of food, solving conflicts, and decision-making. GTO is mathematically modeled as a five-stage algorithm where three stages are responsible for exploration, while the remaining two stages are related to exploitation. The positions of gorillas are updated using the following equations:

(1) $$X\left(t+1\right)=\{\begin{array}{c} \left(U_{l}-L_{l}\;\right)\times r_{1}+L_{l}\;rand<p\\ \left(r_{2}-C\right)\times X_{r}\left(t\right)+L\times H\;rand\;\geq 0.5\\ X\left(t\right)-L\times (L\times \left(X\left(t\right)-X_{r}\left(t\right)\right)+r_{3}\times \left(X\left(t\right)-X_{r}\left(t\right))\right)\;rand<0.5\; \end{array}$$

where X is the position of the current gorilla at iteration t. $r_{1},\;\;r_{2},\;r_{3}$, and rand are random numbers in the range of 0 to 1. p is a parameter whose value will usually lies between 0 and 1. $U_{l}$ and $L_{l}$ are the upper and lower boundaries, respectively. $X_{r}$ is a gorilla randomly chosen at each iteration. The values of *C*, *L*, and *H* are calculated using Equations (2), (4) and (5), respectively.

(2) $$C=F\times \left(1-\frac{Iter}{Maxit}\right)$$

(3) $$F=\cos(2\times r_{4})+1$$

(4) L=C×l

(5) H=Z×X(t)

In Equation (2), Iter represents the current iteration and Maxit represents the maximum number of iterations. F in Equation (2) is calculated using Equation (3), and $r_{4}$ is a random number in the range of 0 to 1. Here, l is an integer randomly chosen in the range of −1 to 1. In Equation (5), Z is a random number in the range −C to +C. Based on the position of the silverback, the other gorillas change their position while searching for food, and this behavior is represented using Equation (6). The M value mentioned in Equation (6) is computed using Equations (7) and (8).

(6) $$X\left(t+1\right)=L\times M\times \left(X\left(t\right)-X_{silverback}\right)+X\left(t\right)$$

(7) $$M={\left({\left|\frac{1}{N}\right|}^{g}\sum_{i=1}^{N}X_{i}\left(t\right)\right)}^{\frac{1}{g}}$$

(8) $$g=2^{L}$$

where $X_{silverback}$ is the position of the silverback gorilla with the best position when compared with the positions of the other gorillas and N is the total number of gorillas. Gorilla behavior in competing to choose adult females is represented using Equation (9).

(9) $$X\left(t+1\right)=X_{silverback}-\left(X_{silverback}\times Q-X\left(t\right)\times Q\right)\times A$$

(10) $$Q=2\times r_{5}-1$$

(11) A=β×E

(12) $$E=\{\begin{array}{c} N_{1}\;rand\;\geq 0.5\\ N_{2}\;rand<0.5\; \end{array}$$

In the above equations, $r_{5}$ and rand are random numbers in the range of 0 to 1, while β is a parameter whose value is crucial in deciding the updated positions of gorillas. $N_{1}$ is a random number in the range decided by the problem dimension, while $N_{2}$ is a random number that follows a normal distribution in the range [0, 1]. Initially, Equation (1) is used to update all the gorilla positions. Then, the silverback gorilla is found in that iteration. After that, the other gorilla positions will be updated based on the silverback gorilla position. If |C|≥1, then the positions of the gorillas are updated using Equation (6); otherwise, they will be updated using Equation (9).

### 3.6. Particle Swarm Optimization

PSO [47] is one of the popular and efficient swarm intelligence-based optimization algorithms. PSO is inspired by the characteristics exhibited by flocks of birds while searching for food. Usually, the population will be initialized randomly and updated in each iteration based ona fitness function. The velocity of each particle is mathematically modeled and updated using Equation (13).

(13) $$v_{i}\left(t+1\right)=w\times v_{i}\left(t\right)+c_{1}\times r_{1}\times \left(p_{i}\left(t\right)-x_{i}\left(t\right)\right)+c_{2}\times r_{2}\times \left(gbest-x_{i}\left(t\right)\right)$$

where $v_{i}\left(t\right)$ stands for the velocity of the *i*th particle in iteration *t*. Three crucial parameters in PSO are *w*, *c_1_*, and *c_2_*. The position of the *i*th particle in iteration *t* is represented as $x_{i}\left(t\right)$; and $p_{i}\left(t\right)$ and gbest represent the personal best and global best particle positions, respectively. $r_{1}$ and $r_{2}$ are random numbers in the range of 0 to 1. The position of each particle is updated based on the old position and new velocity, as represented in Equation (14).

(14) $$x_{i}\left(t+1\right)=x_{i}\left(t\right)+v_{i}\left(t+1\right)$$

The personal best and global best are computed in each iteration using Equations (15) and (16), respectively.

(15) $$p_{i}\left(t+1\right)=\{\begin{array}{c} p_{i}\left(t\right)\;if\;f\left(x_{i}\left(t+1\right)\right)\geq f\left(p_{i}\left(t\right)\right)\\ x_{i}\left(t+1\right)\;if\;f\left(x_{i}\left(t+1\right)\right)<f\left(p_{i}\left(t\right)\right) \end{array}$$

(16) $$\begin{array}{cc} gbest & \in \left\{p_{0}\left(t\right),p_{1}\left(t\right),\;\ldots .,\;p_{m}\left(t\right)\;\right\}\\ & =min\;\left\{f\left(p_{0}\left(t\right)\right),f\left(p_{1}\left(t\right)\right),\;\ldots ,f\left(p_{m}\left(t\right)\right)\;\right\} \end{array}$$

where f represents the fitness function, which is crucial in deciding the performance of PSO.

### 3.7. Elephant Herding Optimization

Elephant Herding Optimization (EHO) [48] is inspired by the behavior of elephants. Like PSO and GTO, EHO also falls under the category of swarm intelligence meta-heuristic algorithms. The position of an elephant is updated using Equation (17).

(17) $$x_{i}^{new}=x_{i}^{old}+\alpha \;\left(x_{best}-x_{i}^{old}\right)*ran\;$$

where $x_{i}^{new}$ and $x_{i}^{old}$ are the new and old positions of the *i*th elephant. $x_{best}$ is the best elephant position found using Equation (18). $x_{center}$ in Equation (18) is computed using Equation (19). In addition to updating the best elephant position, the worst elephant position $x_{worst}$ is also updated using Equation (20).

(18) $$x_{best}=\beta \times x_{center}$$

(19) $$x_{center}=\frac{1}{n}\times \sum_{i=1}^{n}x_{i}$$

(20) $$x_{worst}=x_{min}+\left(x_{max}-x_{min}+1\right)*rand$$

where α and β are the EHO parameters; rand is a random number in the range [0, 1]; and *n* is the number of elephants. $x_{max}$ and $x_{min}$ are the maximum and minimum boundaries for elephant positions.

## 4. Materials and Methods

**Figure 1:**
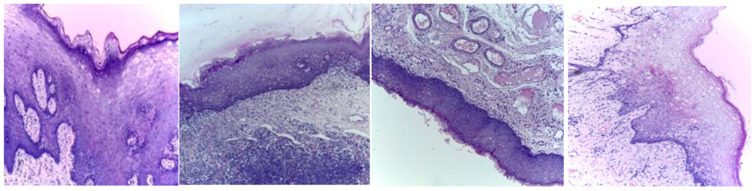
Sample oral histopathological images belonging to the Normal class.

**Figure 2:**
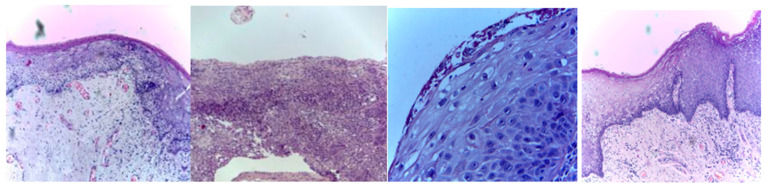
Sample oral histopathological images belonging to OSCC class.

Three publicly available datasets comprising 2784 Normal and 3632 oral squamous cell carcinoma subjects are considered in this work. The first dataset was obtained from Kaggle [49], and it contains oral histopathological images at both the 100× and 400× zoom levels. The first dataset contains a total of 5192 images, and out of them, 2494 images belong to the Normal class and 2698 belong to OSCC class. The second and third datasets are obtained from the online repository built by Tabassum Yesmin Rahman et al. [50]. Oral histopathological images with zoom levels of 100× and 400× are present in the second and third datasets, respectively. A total of 89 normal images and 439 OSCC images are available in the second dataset, while 201 normal images and 495 OSCC images are available in the third dataset. Some of the sample oral histopathological images belonging to normal and OSCC classes are shown in Figure 1 and Figure 2, respectively.

**Figure 3:**
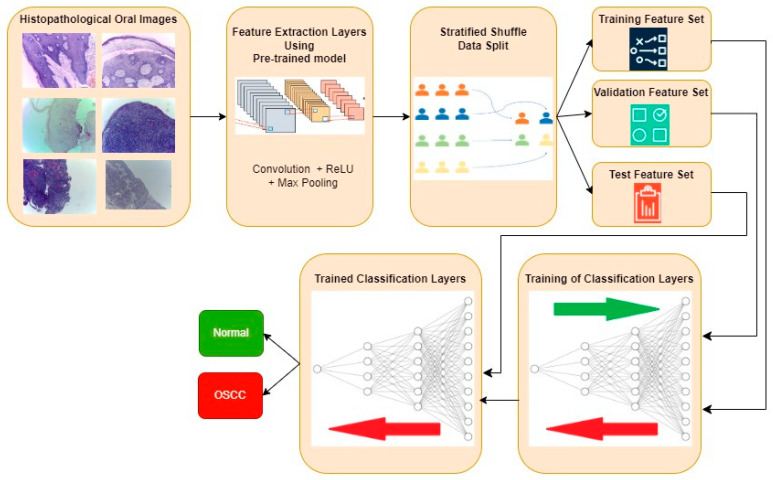
Typical approach for OSCC detection using transfer learning-based feature extraction.

The typical procedure for implementing oral cancer detection using transfer learning-based feature extraction is shown in Figure 3. Histopathological oral images from the three datasets are fed to the feature extraction layers discretely, and the resultant classification performance metrics are also computed individually. Features are extracted using the transfer learning approach, where the weights are pre-trained for another similar dataset. Three popular CNN architectures, namely, InceptionV2, MobileNetV3, and EfficientNetB3, are investigated in this work for feature extraction. Weights that are pre-trained for the popular ImageNet dataset are considered in all three architectures. The extracted features are then divided into training, validation, and test feature sets using a stratified shuffle split approach in a 70:15:15 ratio, respectively. A validation set is used to avoid overfitting and to improve the performance of the model for the unseen new data. A stratified shuffle split is considered since it randomly selects the samples according to the class ratio in the original dataset. In other words, stratified shuffle split ensures the ratio of each class in all three resultant sets is the same as that shown in Table 1. This approach of data splitting is very crucial in imbalanced datasets. Then, the classification layers are trained using training and validation feature sets, where the ideal weights of Neural Networks for classifying the oral histopathological images are found.

**Figure 4:**
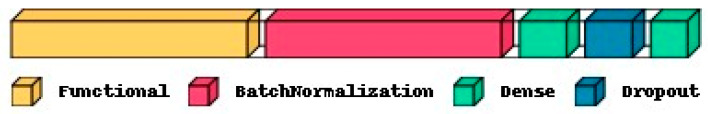
Typical deep learning architecture with a functional layer depicting the transfer learning model for feature extraction and the remaining layers depicting the classification layers.

Two fully connected Neural Network layers along with batch normalization and dropout are used as classification layers, as shown in Figure 4. Finally, the trained classification layers with ideal weights are used to classify the test feature set as Normal or OSCC class. In Figure 4, the functional layer depicts the transfer learning-based pre-trained model while the remaining layers are used for classification. The specifications of the classification layer considered in this research work are presented in Table 2. For comparison purposes, the classification layer is unaltered for all the datasets and different feature extraction layers. Specifications related to the number of epochs and batch size during training, optimizer, early stopping, and a reduction in the learning rate on the plateau are also mentioned in Table 2. Based on the transfer model used for the feature extraction layer, the number of trainable parameters of the complete deep learning model will vary, as shown in Table 3. The number of features extracted per input image with the three different feature extraction layers is also shown in Table 3.

**Figure 5:**
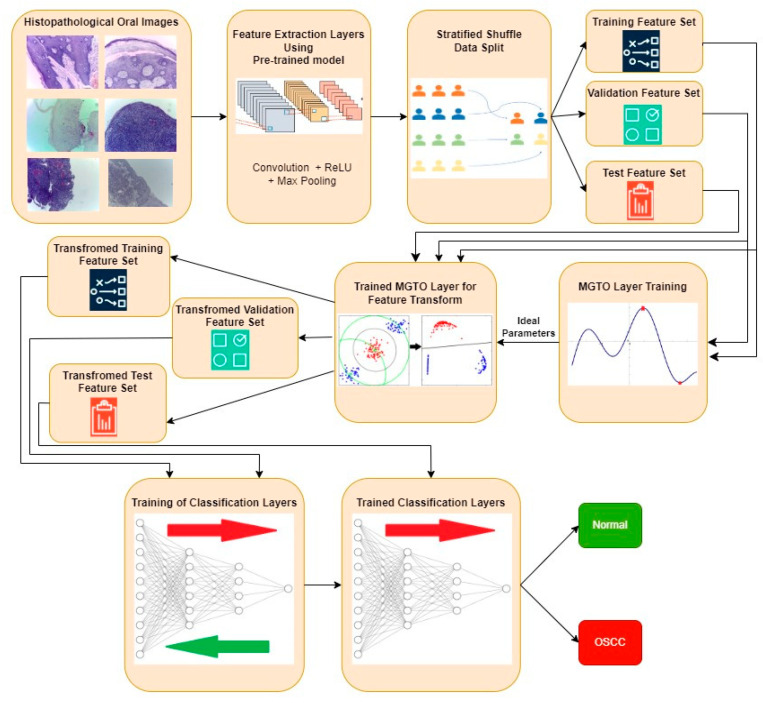
Overview of the proposed approach for OSCC detection.

**Figure 6:**
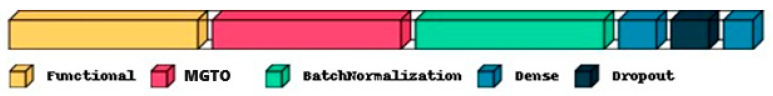
Proposed deep learning architecture where MGTO is used as an intermediate layer between the feature extraction (functional) layer and the classification layer.

The proposed approach for OSCC detection is presented in Figure 5 and Figure 6. An intermediate layer based on MGTO is included in the proposed method when compared with Figure 3 and Figure 4. Similar to the classification layer, the newly introduced intermediate layer also needs to be trained, where it will learn the ideal values for its parameters related to the MGTO algorithm. Hence, it is trained with the original training and validation feature sets. Then, all three original feature sets are supplied as input to the trained MGTO layer, where the feature sets are transformed to produce another three transformed sets, namely, training, testing, and validation. The size of the input and output feature sets remains the same. Then, the transformed sets are considered classification layers for detecting the class of an oral histopathological image.

## 5. Implementation of the Proposed MGTO

The equations to update the positions of gorillas are modified based on the Sine Cosine Algorithm 1 [51] to increase the exploitation and exploration capabilities of GTO. In MGTO, three equations that update the positions of gorillas are modified. Equations (1), (6) and (9) of GTO are modified, as represented in Equations (21)–(23), respectively, in MGTO. All other equations of GTO remain intact in MGTO.

(21) $$X\left(t+1\right)=\{\begin{array}{c} \left(U_{l}-L_{l}\;\right)\times r_{1}+L_{l}\;rand<p\\ \left(r_{2}-C\right)\;X_{r}\left(t\right)+L\times H\;rand\;\geq 0.5\\ X\left(t\right)-L\times rad\times sin\left(X\left(t\right)-X_{r}\left(t\right)\right)+rad\times cos\left(X\left(t\right)-X_{r}\left(t\right))\right)\;rand<0.5\; \end{array}$$

(22) $$X\left(t+1\right)=L\times M\times rad\times sin\left(X\left(t\right)-X_{silverback}\right)+X\left(t\right)$$

(23) $$X\left(t+1\right)=X_{silverback}-rad\times cos\left(X_{silverback}\times Q-X\left(t\right)\times Q\right)\times A\;$$

where rad in the above equations is computed using Equation (24). const in Equation (24) is a constant, and it is considered to be three, as suggested in [48]. Crnt_ Iter represents the current iteration number, while Max_Iter represents the maximum number of iterations.

(24) $$\mathrm{rad}\;=\;\mathrm{const}-Crnt\_\mathrm{Iter}\;\times \left(\frac{\mathrm{const}}{{\mathrm{Max}}_{\mathrm{Iter}}}\right)\;$$

In the original GTO, the gorilla population is initialized randomly. When using MGTO as an intermediate layer in deep learning models, the gorilla population is initialized with the features extracted from the previous layer. The number of gorillas is equal to the number of features extracted. Then, the gorillas’ positions are updated in each iteration using MGTO equations. The fitness function is very crucial in optimization algorithms, and it is selected based on the problem to be solved. To use MGTO for transforming the features, the fitness function based on the variance metric is considered. The fitness of each gorilla, $F\left(X_{i}\right)$ depends on its own position and four nearest-neighbor gorillas, as shown in Equation (25).

(25) $$F\left(X_{i}\right)=\;\mathrm{Variance}\left(X_{i-2},X_{i-1},X_{i},X_{i+1},X_{i+2}\right)\;$$

**Figure 7:**
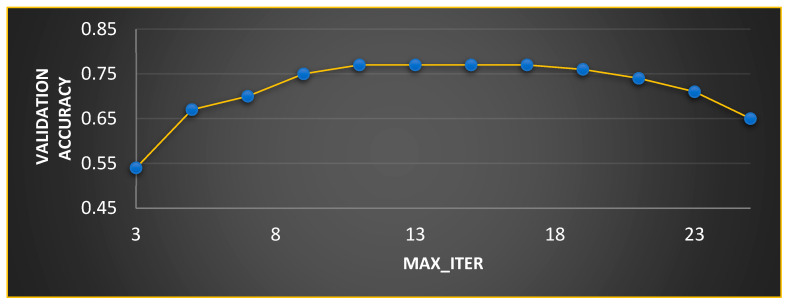
Ideal value computation for Max_Iter.

**Figure 8:**
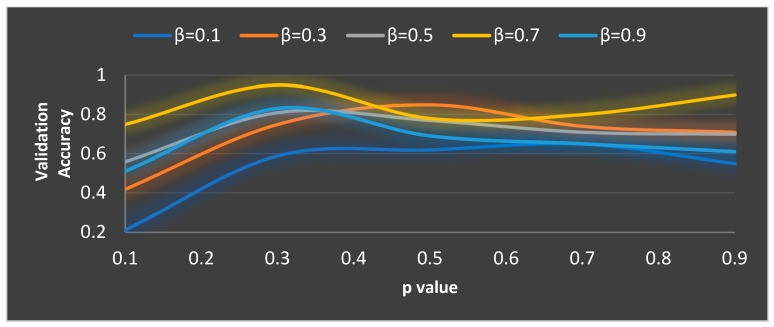
Ideal value computation of the p and β parameters.

In MGTO, p and β are the parameters that mainly determine the performance along with Max_Iter. The ideal values of these parameters are found based on the accuracy attained during training and validation. The validation accuracy for various values of Max_Iter is plotted in Figure 7 and Figure 8. The ideal value is found where the validation accuracy of 0.77 is reached. While finding the optimal value for Max_Iter, the other two parameters, namely, p and β are kept at 0.5 (median of range [0, 1]). To find the optimal values for p and β parameters, Max_Iter is kept at its ideal value of 11. Figure 8 depicts the validation accuracy for various values of the p and β parameters. The highest validation accuracy of 0.95 is attained when p = 0.3 and β = 0.7. Finding the ideal values for the parameters of MGTO is termed training, and for this purpose, training and validation feature sets are used. After training, an MGTO transform is implemented for all three feature sets, namely, the training, validation, and test sets with the ideal parameter values of Max_Iter=11, p = 0.3, and β = 0.7. Notably, these are the ideal parameters of MGTO for the first dataset when MobileNetV3 is used as a feature extraction layer. The ideal parametric values may change depending on the input data given to the MGTO layer. The ideal values for other input data will be presented in the next section. The procedure for implementing MGTO as an intermediate feature transform layer for the test feature set is summarized in Algorithm 1.
**Algorithm 1**: Algorithm to implement the proposed MGTO as an intermediate layer in deep learning models for feature transformation of a test feature set.Step 1: Extract features using pre-trained transfer learning models for each oral histopathological image. Step 2: Consider the number of features as the size of the population in MGTO. Initialize the position of gorillas with extracted features. Step 3: Initialize parameters of MGTO: $U_{l}=\max\left(X^{1},X^{2},\ldots .,X^{N}\right)$*,*
$L_{l}=\min\left(X^{1},X^{2},\ldots .,X^{N}\right)$*,*
${\mathrm{Max}}_{\mathrm{Iter}}=11,$ p = 0.3, and β = 0.7. Step 4: Compute the fitness value of each gorilla using Equation (25). Step 5: Update the position of each gorilla using Equation (21). Step 6: Identify the silverback gorilla, i.e., the gorilla with the highest fitness. Step 7: Update the position of each gorilla using Equation (22) if |C|≥1. Otherwise, use Equation (23). Step 8: Repeat steps 4 to 7 until the maximum number of iterations is reached. If the maximum number of iterations are completed, then go to step 9. Step 9: Consider the final position of the gorillas as the output of the feature transform and give them as input to the classification layer.

## 6. Results and Discussion

**Figure 9:**
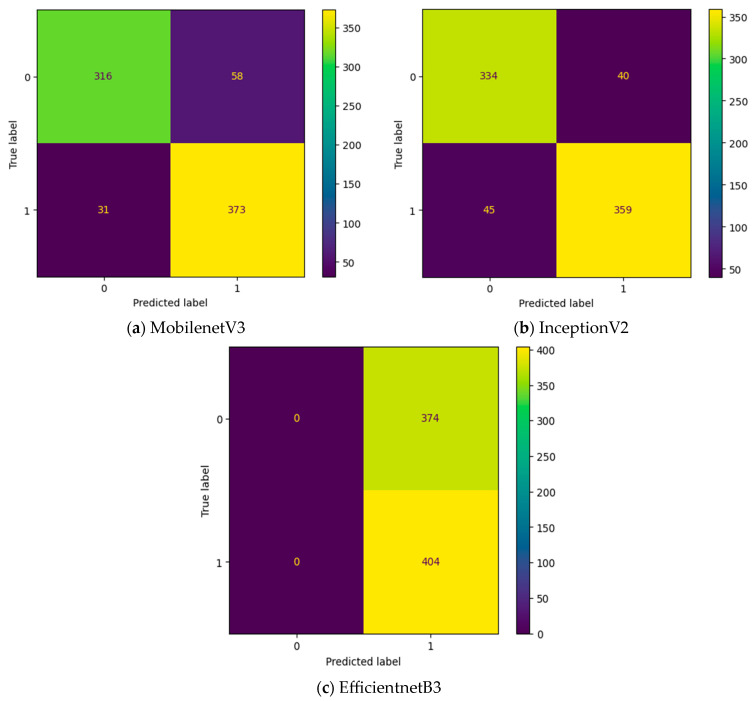
Confusion matrix of deep learning models without an intermediate layer.

Initially, the experiment is conducted without any intermediate layers in the deep learning model. Three different transfer learning-based models, namely, InceptionV2, MobileNetV3, and EfficientNetB3, are tested as feature extraction layers. As mentioned in Table 2, the specifications of the classification layer remain the same for all three different feature extraction layers. The confusion matrix attained for these three deep learning models without an intermediate layer on the first dataset in OSCC detection is shown in Figure 9. In Figure 9, the label 0 represents the Normal class and label 1 represents class OSCC class. EfficientNetB3 classifies all the input images as OSCC, and so its True Negative (TN) = 0. This clearly indicates the poor performance of the EfficientNetB3-based deep learning model without an intermediate layer. To detect OSCC, a high TP is required, while to detect the Normal class properly, a high TN is required. Among the remaining two models without an intermediate layer, the highest True Positive (TP) is attained with MobileNetV3, while the highest TN is attained with InceptionV2.

**Figure 10:**
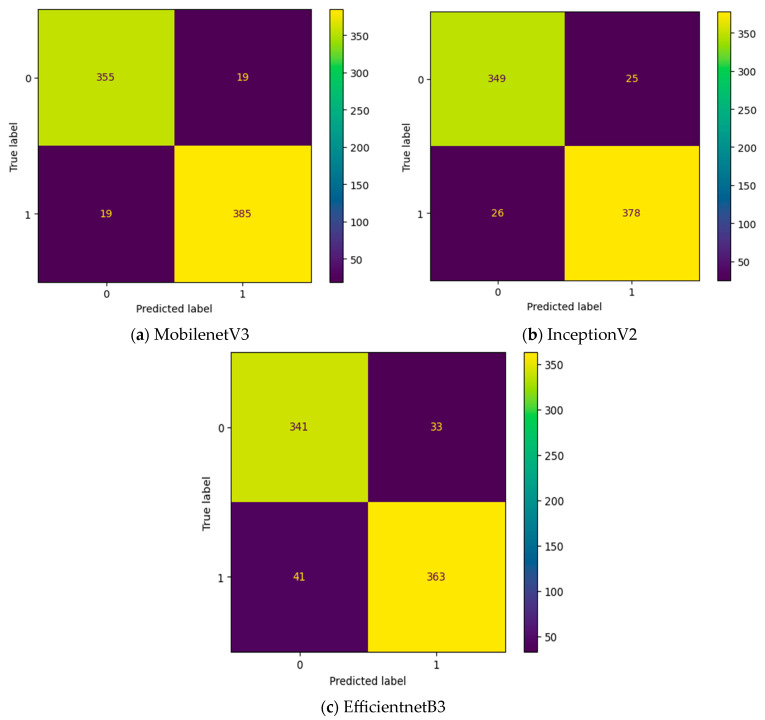
Confusion matrix of deep learning models with MGTO as an intermediate layer.

To improve the number of TNs and TPs, an MGTO-based intermediate layer is proposed in this work. Figure 10 shows the confusion matrix of three different feature extraction-based deep learning models with MGTO as the intermediate layer in OSCC detection. When MGTO is not used as an intermediate layer in the EfficientNetB3-based deep learning model, all the oral images are classified as OSCC, while better TN and TP values are attained with the proposed layer. The highest TN and TP values are attained for the proposed MobileNetV3-based feature extraction with MGTO as an intermediate layer.

Based on the confusion matrix, four popular performance metrics, namely, accuracy, precision, recall, and the F1-score, are used in this research work to analyze the performance of the deep learning models. In addition to the deep learning models with and without an MGTO-based intermediate layer, three other swarm intelligence-based optimization algorithms, namely, PSO, EHO, and GTO, are also tested as intermediate layers, and their results are also presented in Table 4. The implementation procedure for PSO, EHO, and GTO as intermediate layers follows Algorithm 1 presented in the previous section. Only the parameters and the way of updating the position of the swarm vary based on the optimization algorithm used. The final ideal parameters of all four tested intermediate layers after training are listed in Table 5.

The main objective of this work is to detect OSCC, so precision, recall, and the F1-score in Table 4 are related to the correct identification of OSCC class, while the accuracy metric is related to the correct identification of both the Normal and OSCC classes. As seen in Table 4, the deep learning models without an intermediate layer provide less accuracy than the proposed deep learning models with MGTO as an intermediate layer.

**Figure 11:**
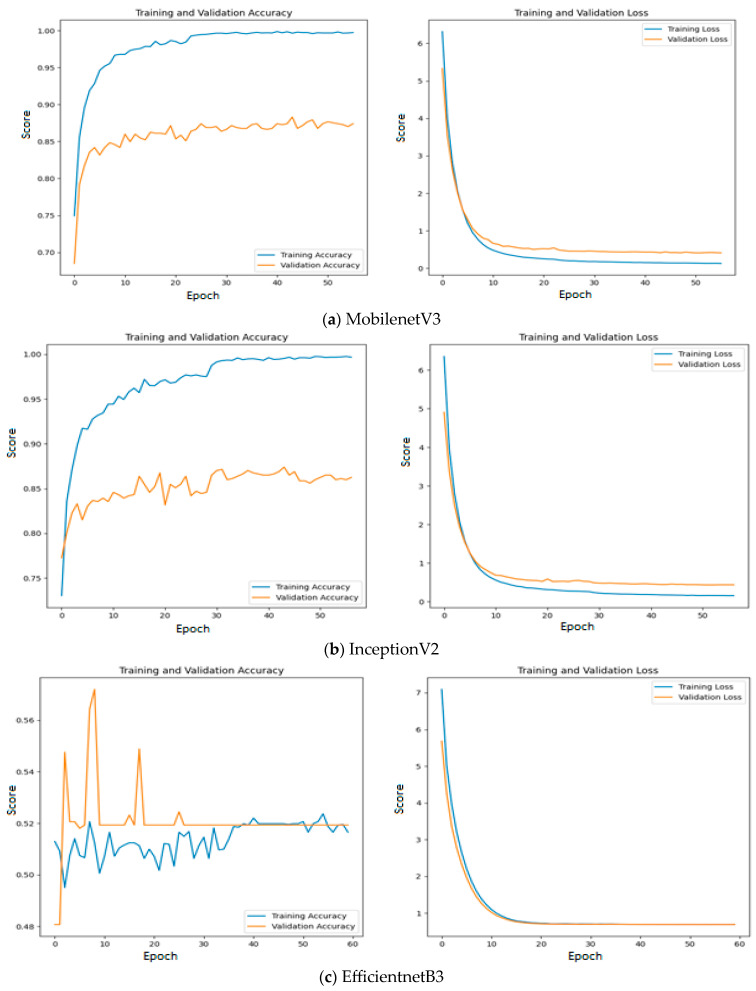
Training and validation accuracy and loss of deep learning models without an intermediate layer on the first dataset.

Among the models without an intermediate layer, MobileNetV3 offers the highest accuracy of 0.89, which is followed by InceptionV2 with an accuracy of 0.88 and EfficientNetB3 with an accuracy of 0.52. The reason for such poor performance of EfficientNetB3 is explained as follows: All three feature extraction models are pre-trained on the ImageNet dataset and features are extracted based on the weights appropriate for the ImageNet dataset. The weights and architecture of EfficientNetB3 fail to capture the significant features from input oral images, while vital features are properly extracted using the remaining two feature extraction models. This statement is further supported by Figure 11, where the training and validation accuracy and loss are presented for all the three investigated deep learning models without an intermediate layer on the first dataset.

**Figure 12:**
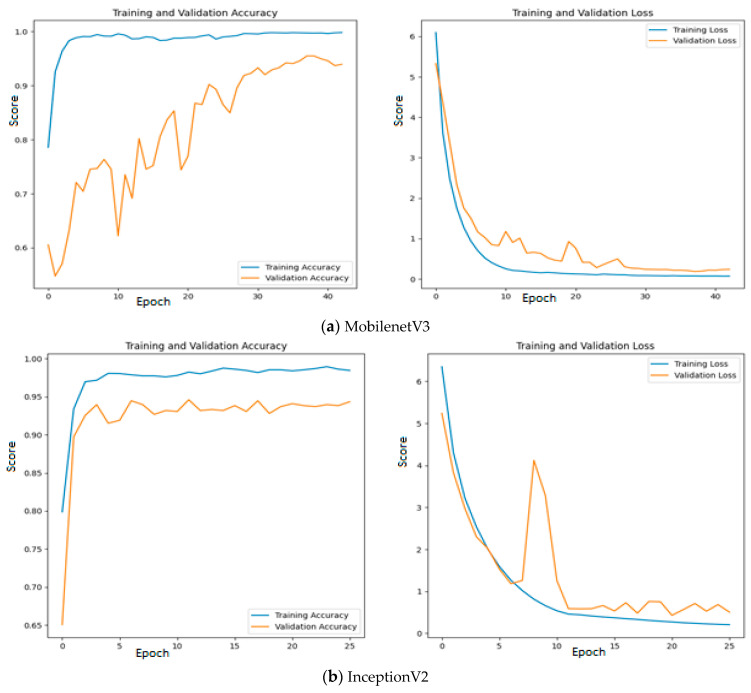

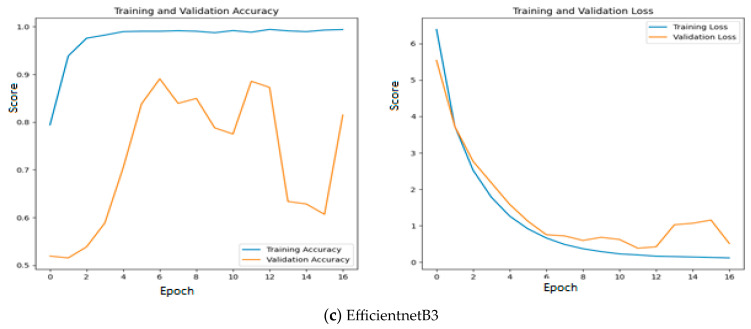
Training and validation accuracy and loss of deep learning models with MGTO as an intermediate layer on the first dataset.

Since quality features are extracted with MobileNetV3 and InceptionV2, both training and validation accuracy increase gradually during training. In addition, both training and validation loss also decrease exponentially. But a deep learning model that uses EfficientNetB3 fails to increase in both training and validation accuracy due to the poor features that are extracted from the histopathological oral images. Figure 12 presents the training and validation accuracy and loss when MGTO is used as an intermediate layer on the first dataset. It clearly shows an improved accuracy during both training and validation because of the transformed appropriate features produced with MGTO. To support the findings based on the first dataset, the other two smaller OSCC datasets are tested. The second and third datasets are highly imbalanced since the number of OSCC class samples is much higher than the number of normal class samples. The performance metrics attained on those two datasets are presented in Table 6 and Table 7.

**Figure 13:**
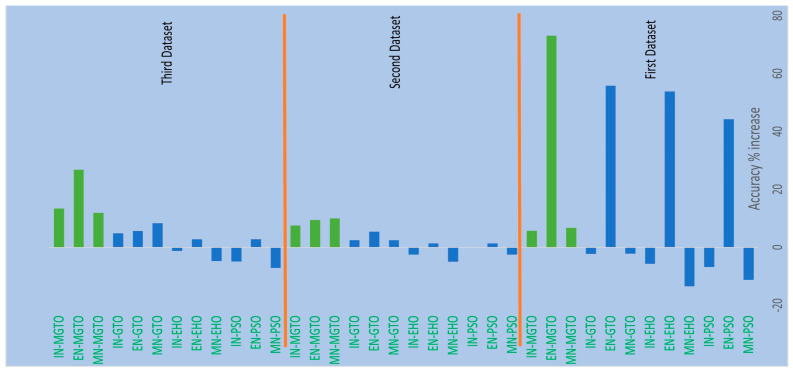
Percentage accuracy increase due to the usage of intermediate layers in DL models when compared with the accuracy offered by DL models without intermediate layer.

From Table 4, Table 6 and Table 7, it is very clear that MGTO works very well as an intermediate layer when compared with the other tested intermediate layers in all three datasets. The significance of MGTO as an intermediate layer can be clearly seen in Figure 13, where the percentage of accuracy increase attained with the usage of various intermediate layers when compared with a deep learning model without an intermediate layer is depicted.

On all three datasets, the percentage of accuracy increase is much less or even negative when PSO, EHO, and GTO are used as intermediate layers on the features extracted from MobileNetV3 and InceptionNetV2. Notably, already these two feature extraction models without an intermediate layer produce an accuracy of more than 0.8 in all three datasets. Implicitly, these intermediate layers fail to significantly improve the accuracy since they are not able to produce more appropriate transformed features for classification. Only for the features extracted with EfficientNetB3 from the first dataset can these intermediate layers provide a significant accuracy increase. This is because the original features extracted with EfficientNetB3 are very poor on the first dataset, which yields an accuracy of only 0.52. Out of these three intermediate layers, GTO comparatively performs well on all three datasets. Hence evidence for improving GTO further with suitable modifications was found. MGTO is formulated with the modifications stated in the previous section, and it worked well on all three datasets.

**Figure 14:**
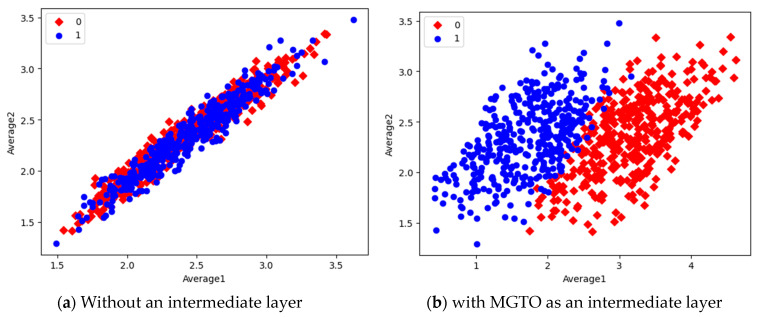
Scatter plot of features extracted with MobileNetV3.

**Figure 15:**
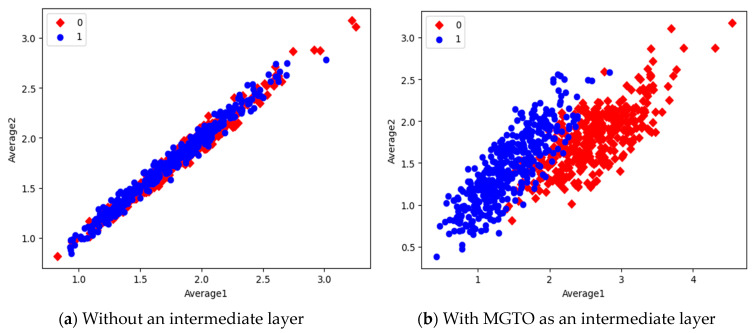
Scatter plot of features extracted with InceptioNetV2.

**Figure 16:**
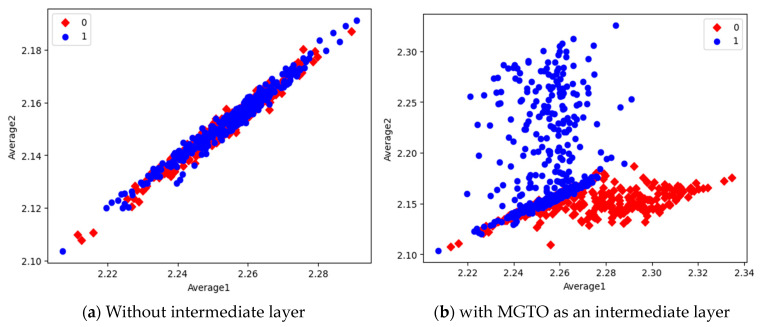
Scatter plot of features extracted by EfficientNetB3.

In the first dataset, a 73% increase in accuracy is seen using the EfficientNetB3-based DL model due to the usage of MGTO as an intermediate layer. Nearly a 6% accuracy increase is seen due to the usage of MGTO in the MobileNetV3 and InceptionV2-based DL models. Notably, the highest accuracy of 0.95 is produced on the first dataset using the MobileNetV3-MGTO-based DL model. Even on the imbalanced second and third datasets, MGTO is capable of producing significant increases in accuracy. The reason for this better performance is threefold. Firstly, the modification of GTO with the Sine and Cosine algorithm increases its exploration and exploitation capability well. Exploitation is responsible for the local search, i.e., fine-tuning and exploration are responsible for the global search. Secondly, the selection of appropriate fitness functions. Local variance-based fitness functions worked well to transform the features of different classes in different ways. Thirdly, the usage of ideal parameters in MGTO resulted in better accuracy. As shown in Figure 7 and Figure 8, the values of MGTO parameters have a huge impact on accuracy. Due to the above-mentioned reasons, MGTO works soundly as an intermediate layer that transforms the input features into more appropriate features for classification. In other words, the introduction of the proposed intermediate layer helps the classifier to distinguish the features of two different classes. This statement is backed by the scatter plots shown in Figure 14, Figure 15 and Figure 16. In the scatter plots, label 0 represents the Normal class and label 1 represents the OSCC class. To represent the features of the first dataset in a scatter plot, two averages are computed. Average1 is the mean of the first half of the features and Average2 is the mean of the remaining half of the features. For example, 1280 features are extracted with MobileNetV3, and the mean of the first 640 features is considered as Average1, and the mean of the remaining 640 features is considered as Average2.

In the scatter plots, the MobileNetV3 features of two the classes are slightly scattered and overlapped, while the EfficientNetB3 features are heavily overlapped. A comparison of the scatter plots with and without intermediate layers for all three feature extraction models suggests the significance of MGTO. The proposed layer transforms the features in a manner that is more suitable for classification by spreading the two different class features apart to some extent. When these transformed features are used for training and validation, the classification layer is trained well. Finally, better performance is achieved when the transformed test dataset is categorized by the trained classification layer.

**Figure 17:**
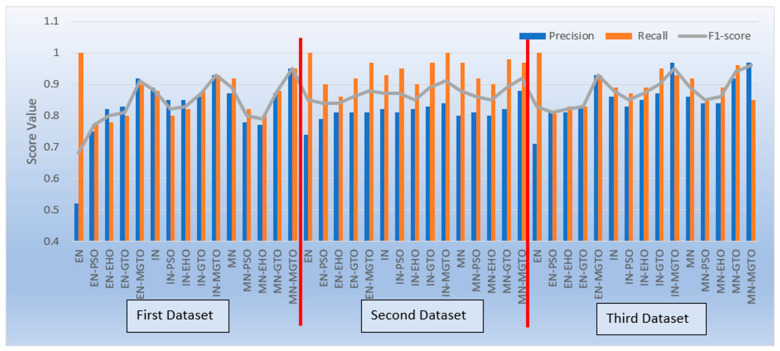
Precision, recall, and F1-score of various OSCC classification models.

Apart from accuracy, other performance metrics are also relevantly important. Precision gives the percentage of correct OSCC predictions among the total number of OSCCs predicted. Recall is related to the percentage of actual OSCC that was identified correctly as OSCC. The F1-score is the harmonic mean of precision and recall. These metrics are depicted for all three datasets in Figure 17. Considering these three metrics, DL models with MGTO as an intermediate layer outperform all other investigated intermediate layers. In the first dataset, the highest performance is offered by the proposed MobileNetV3-MGTO-based DL model, which achieves precision = 0.95, recall = 0.95, and F1-score = 0.95. Even on the second and third datasets, the highest F1-score is archived with the proposed DL model. Though the highest F1-score and accuracy are attained with the proposed DL model on all three datasets, it fails to attain balanced precision and recall in imbalanced datasets. For example, recall is much higher than precision for the proposed DL model on the second dataset, while precision is much higher than recall for the proposed DL model on the third dataset. But it attains almost equal precision and recall on the first dataset, which is a balanced dataset. Hence, wherever higher values of both precision and recall are required on imbalanced datasets, the proposed DL model underperforms the other models. This could be considered as the first limitation of the proposed model.

**Figure 18:**
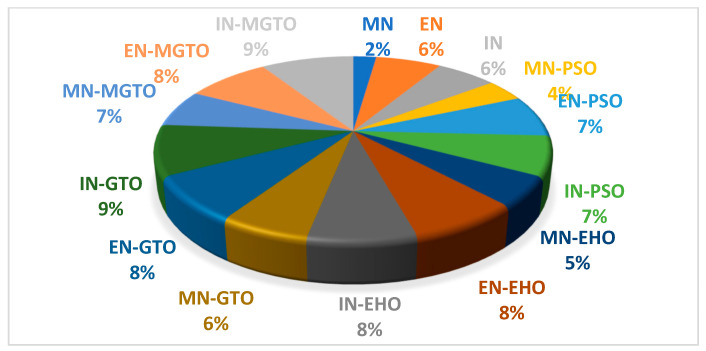
Pie-chart representing the percentage of training time taken by each DL model with respect to total training time taken by all DL models.

The training time for all the investigated DL models on the first dataset is presented in Table 8. DL models without an intermediate layer are trained comparatively quickly, while the presence of an intermediate layer may take more training time [52]. MobileNetV3 has a shorter training time since the number of features extracted is 1280, while the number of features extracted with EfficientNetB3 and InceptionNetV2 is 1536. A pie chart is presented in Figure 18 that depicts the percentage of training time required by a DL model when compared with the total training time required by all the DL models. PSO and EHO take relatively less training time than other intermediate layers due to their simple structure. When compared with GTO, the proposed MGTO intermediate layer takes more training time due to the inclusion of Sine and Cosine argument calculations. Only 2% of the total training time is taken by the MobileNetV3 DL model without an intermediate layer, while 7% of the total training time is taken by the proposed DL model. This could be considered as second limitation.

An accuracy comparison of some related works for oral cancer detection is presented in Table 9. Supervised classifiers such as K-Nearest Neighbor, and Support Vector machine attain comparatively lower accuracy than the deep learning models. The proposed deep learning model with MGTO as the intermediate layer offers the highest accuracy of 95%, which shows the importance of the proposed DL model.

## 7. Conclusions

This research work focuses on developing an enhanced deep-learning model to diagnose OSCC disease. The proposed DL model with MGTO as the intermediate layer and MobileNetV3 as the feature extraction layer could classify 95% of the histopathological oral images correctly. Three oral histopathological image datasets were tested and on all three datasets, and the inclusion of MGTO as an intermediate layer enhanced the accuracy of the DL model. Features were transformed using MGTO to produce more appropriate features for classification. MGTO outperformed other investigated SI algorithms as an intermediate layer when compared with PSO, EHO, and GTO, primarily due to the modifications made to the GTO equations and their fitness function. The limitations of the proposed DL model are a relatively higher training time and loss of either precision or recall scores on an imbalanced dataset. Ouruture work will investigate other SI algorithms as intermediate layers in DL models. In addition, the proposed model needs to be tested for other medical image classification problems.

## Figures and Tables

**Table 1:** Stratified shuffle data split into three datasets.

Dataset	Class	Total Number of Samples	Number of Training Samples	Number of Validation Samples	Number of Test Samples
First	Normal	2494	1746	374	374
	OSCC	2698	1890	404	404
Second	Normal	89	63	13	13
	OSCC	439	307	66	66
Third	Normal	201	141	30	30
	OSCC	495	347	74	74

**Table 2:** Specifications of the classification layers and techniques used.

Classification Layers and Techniques Used	Specifications
Batch Normalization	momentum = 0.99, epsilon = 0.001
Dense	units = 256, kernel regularizer = L2 regularizer with coefficient L = 0.016, activity regularizer = L1 regularizer with coefficient L = 0.006, bias regularizer = L1 regularizer with coefficient L = 0.006, activation = ReLu
Dropout	drop rate = 0.45
Dense	units = 2, activation = SoftMax
Training	epochs = 100, batch size = 128, stratified shuffle split: training—70%, testing—15%, validation—15%
Optimizer	Adamax with learning rate = 0.001, loss = sparse categorical cross-entropy, metrics = accuracy
Early stopping	patience = 5, minimum delta = 0, monitor = validation loss, restore best weights = true, mode = minimum
Reduce learning rate on plateau	monitor = validation loss, factor = 0.2, patience = 4, mode = minimum

**Table 3:** Specifications of the feature extraction layers.

Feature Extraction Layers	Total Number of Parameters	Number of Trainable Parameters	The Number of Features Extracted
Mobilenet V3	2,591,554	331,010	1280
Efficientnet B3	11,183,665	397,058	1536
InceptionV2	54,736,866	397,058	1536

**Table 4:** Performance metrics computed on the test set of the first dataset.

Transfer Learning Model	Intermediate Layer	Accuracy	Precision	Recall	F1-Score
MobilenetV3	NO	0.89	0.87	0.92	0.89
EfficientnetB3	NO	0.52	0.52	1	0.68
InceptionV2	NO	0.88	0.89	0.88	0.88
MobilenetV3	PSO	0.79	0.78	0.82	0.8
EfficientnetB3	PSO	0.75	0.75	0.78	0.77
InceptionV2	PSO	0.82	0.85	0.8	0.82
MobilenetV3	EHO	0.77	0.77	0.8	0.79
EfficientnetB3	EHO	0.8	0.82	0.78	0.8
InceptionV2	EHO	0.83	0.85	0.82	0.83
MobilenetV3	GTO	0.87	0.87	0.88	0.88
EfficientnetB3	GTO	0.81	0.83	0.8	0.81
InceptionV2	GTO	0.86	0.86	0.88	0.87
MobilenetV3	MGTO	0.95	0.95	0.95	0.95
EfficientnetB3	MGTO	0.9	0.92	0.9	0.91
InceptionV2	MGTO	0.93	0.93	0.93	0.93

**Table 5:** Ideal parameters of various intermediate layers.

Feature Extraction	Intermediate Layer	Ideal Parameter Values
MobilenetV3	PSO	Max_Iter = 10, *w* = 0.6, *c*1 = 0.7, and *c*2 = 0.9
	EHO	Max_Iter = 12, α = 0.9, and β = 0.8
	GTO	Max_Iter=11, p = 0.2, and β = 0.7
	MGTO	Max_Iter=11, p = 0.3, and β = 0.7
EfficientnetB3	PSO	Max_Iter = 12, *w* = 0.4, *c*1 = 0.7, and *c*2 = 0.9
	EHO	Max_Iter = 12, α = 0.7, and β = 0.8
	GTO	Max_Iter=8, p = 0.5, and β = 0.7
	MGTO	Max_Iter=9, p = 0.3, and β = 0.8
InceptionV2	PSO	Max_Iter = 12, *w* = 0.6, *c*1 = 0.8, and *c*2 = 0.8
	EHO	Max_Iter = 11, α = 0.8, and β = 0.6
	GTO	Max_Iter=7, p = 0.4, and β = 0.7
	MGTO	Max_Iter=9, p = 0.4, and β = 0.6

**Table 6:** Performance metrics computed on the test set of the second dataset.

Transfer Learning Model	Intermediate Layer	Accuracy	Precision	Recall	F1-Score
MobilenetV3	NO	0.8	0.8	0.97	0.88
EfficientnetB3	NO	0.74	0.74	1	0.85
InceptionV2	NO	0.8	0.82	0.93	0.87
MobilenetV3	PSO	0.78	0.81	0.92	0.86
EfficientnetB3	PSO	0.75	0.79	0.9	0.84
InceptionV2	PSO	0.8	0.81	0.95	0.87
MobilenetV3	EHO	0.76	0.8	0.9	0.85
EfficientnetB3	EHO	0.75	0.81	0.86	0.84
InceptionV2	EHO	0.78	0.82	0.9	0.85
MobilenetV3	GTO	0.82	0.82	0.98	0.89
EfficientnetB3	GTO	0.78	0.81	0.92	0.86
InceptionV2	GTO	0.82	0.83	0.97	0.89
MobilenetV3	MGTO	0.88	0.88	0.97	0.92
EfficientnetB3	MGTO	0.81	0.81	0.97	0.88
InceptionV2	MGTO	0.86	0.84	1	0.91

**Table 7:** Performance metrics computed on the test set of the third dataset.

Transfer Learning Model	Intermediate Layer	Accuracy	Precision	Recall	F1-Score
MobilenetV3	NO	0.84	0.86	0.92	0.89
EfficientnetB3	NO	0.71	0.71	1	0.83
InceptionV2	NO	0.82	0.86	0.89	0.88
MobilenetV3	PSO	0.78	0.84	0.85	0.85
EfficientnetB3	PSO	0.73	0.81	0.81	0.81
InceptionV2	PSO	0.78	0.83	0.87	0.85
MobilenetV3	EHO	0.8	0.84	0.89	0.86
EfficientnetB3	EHO	0.73	0.81	0.83	0.82
InceptionV2	EHO	0.81	0.85	0.89	0.87
MobilenetV3	GTO	0.91	0.92	0.96	0.94
EfficientnetB3	GTO	0.75	0.83	0.83	0.83
InceptionV2	GTO	0.86	0.87	0.95	0.9
MobilenetV3	MGTO	0.94	0.97	0.85	0.96
EfficientnetB3	MGTO	0.9	0.93	0.93	0.93
InceptionV2	MGTO	0.93	0.97	0.93	0.95

**Table 8:** Time taken to train various DL models.

DL Model	Training Time (hh:mm:ss)	DL Model	Training Time (hh:mm:ss)
MN	00:05:33	IN-EHO	00:18:22
EN	00:15:42	MN-GTO	00:15:15
IN	00:14:37	EN-GTO	00:19:17
MN-PSO	00:09:23	IN-GTO	00:21:39
EN-PSO	00:17:52	MN-MGTO	00:15:52
IN-PSO	00:17:01	EN-MGTO	00:20:12
MN-EHO	00:12:47	IN-MGTO	00:22:21
EN-EHO	00:18:46		

**Table 9:** Comparison of the accuracy attained in related works.

Related Work	Year	Classification Framework	Accuracy (%) Attained
Rahman A.U. et al. [27]	2022	AlexNet	90.06%
Aberville M. [52]	2017	Convolutional Neural Network	88.3%
Alkhadar H. [53]	2021	KNN, Logistic Regression, Decision Tree, Random Forest	76%
Alhazmi A. [54]	2021	Artificial Neural Network	78.95%
Chu C.S. [55]	2020	SVM, KNN	70.59%
Welikala R.A. [56]	2020	ResNet101	78.30%
Shavlokhova V. [57]	2021	CNN	77.89%
Proposed	2023	Pre-trained MobileNetV3 for feature extraction and MGTO as an intermediate layer	95%

## Data Availability

The data used for the findings will be shared by the author upon request.

## References

[1] Gupta B., Bray F., Kumar N., Johnson N.W. (2017). Associations between oral hygiene habits, diet, tobacco and alcohol and risk of oral cancer: A case–control study from India. Cancer Epidemiol..

[2] Ramakrishna M.T., Venkatesan V.K., Izonin I., Havryliuk M., Bhat C.R. (2023). Homogeneous Adaboost Ensemble Machine Learning Algorithms with Reduced Entropy on Balanced Data. Entropy.

[3] Laprise C., Shahul H.P., Madathil S.A., Thekkepurakkal A.S., Castonguay G., Varghese I., Shiraz S., Allison P., Schlecht N.F., Rousseau M.C. (2016). Periodontal diseases and risk of oral cancer in Southern India: Results from the HeNCe Life study. Int. J. Cancer.

[4] Khayatan D., Hussain A., Tebyaniyan H. (2023). Exploring animal models in oral cancer research and clinical intervention: A critical review. Vet. Med. Sci..

[5] Mosaddad S.A., Beigi K., Doroodizadeh T., Haghnegahdar M., Golfeshan F., Ranjbar R., Tebyanian H. (2021). Therapeutic applications of herbal/synthetic/bio-drug in oral cancer: An update. Eur. J. Pharmacol..

[6] Borse V., Konwar A.N., Buragohain P. (2020). Oral cancer diagnosis and perspectives in India. Sens. Int..

[7] Ajay P., Ashwinirani S., Nayak A., Suragimath G., Kamala K., Sande A., Naik R. (2018). Oral cancer prevalence in Western population of Maharashtra, India, for a period of 5 years. J. Oral. Res. Rev..

[8] Karadaghy O.A., Shew M., New J., Bur A.M. (2019). Development and assessment of a machine learning model to help predict survival among patients with oral squamous cell carcinoma. JAMA Otolaryngol. Head Neck Surg..

[9] Seoane-Romero J., Vazquez-Mahia I., Seoane J., Varela-Centelles P., Tomas I., Lopez-Cedrun J. (2012). Factors related to late stage diagnosis of oral squamous cell carcinoma. Med. Oral Patol. Oral Cir. Bucal.

[10] Dascălu I.T. (2018). Histopathological aspects in oral squamous cell carcinoma. J. Dent. Sci..

[11] Mangalath U., Mikacha M.K., Abdul Khadar A.H., Aslam S., Francis P., Kalathingal J. (2014). Recent trends in prevention of oral cancer. J. Int. Soc. Prev. Community Dent..

[12] O’Mahony N., Campbell S., Carvalho A., Harapanahalli S., Hernandez G.V., Krpalkova L., Riordan D., Walsh J. (2019). Deep learning vs. traditional computer vision. Science and Information Conference.

[13] Hussein I.J., Burhanuddin M.A., Mohammed M.A., Benameur N., Maashi M.S., Maashi M.S. (2021). Fully automatic identification of gynaecological abnormality using a new adaptive frequency filter and histogram of oriented gradients (hog). Expert. Syst..

[14] Sun Y., Xue B., Zhang M., Yen G.G. (2020). Completely Automated CNN Architecture Design Based on Blocks. IEEE Trans. Neural Netw. Learn. Syst..

[15] Johner F.M., Wassner J. Efficient evolutionary architecture search for CNN optimization on GTSRB. Proceedings of the 18th IEEE International Conference on Machine Learning and Applications.

[16] Mozafari M., Farahbakhsh R., Crespi N. (2020). A BERT-Based Transfer Learning Approach for Hate Speech Detection in Online Social Media. Stud. Comput. Intell..

[17] Khoh W.H., Pang Y.H., Teoh A.B.J., Ooi S.Y. (2021). In-air hand gesture signature using transfer learning and its forgery attack. Appl. Soft Comput..

[18] Mirjalili S., Mirjalili S.M., Lewis A. (2014). Grey Wolf Optimizer. Adv. Eng. Softw..

[19] Krishnan M.M.R., Chakraborty C., Ray A.K. (2010). Wavelet based texture classification of oral histopathological sections. Int. J. Microsc. Sci. Technol. Appl. Educ..

[20] Krishnan M.M.R., Shah P., Choudhary A., Chakraborty C., Paul R.R., Ray A.K. (2011). Textural characterization of histopathological images for oral sub-mucous fibrosis detection. Tissue Cell.

[21] Krishnan M., Acharya U., Chakraborty C., Ray A. (2011). Automated diagnosis of oral cancer using higher order spectra features and local binary pattern: A comparative study. Technol. Cancer Res. Treat..

[22] Patra R., Chakraborty C., Chatterjee J. (2012). Textural analysis of spinous layer for grading oral submucous fibrosis. Int. J. Comput. Appl..

[23] Krishnan M.M.R., Venkatraghavan V., Acharya U.R., Pal M., Paul R.R., Min L.C., Ray A.K., Chatterjee J., Chakraborty C. (2012). Automated oral cancer identification using histopathological images: A hybrid feature extraction paradigm. Micron.

[24] Thomas B., Kumar V., Saini S. Texture analysis based segmentation and classification of oral cancer lesions in color images using ANN. Proceedings of the 2013 IEEE International Conference on Signal Processing, Computing and Control (ISPCC).

[25] Rahman T., Mahanta L., Chakraborty C., Das A., Sarma J. (2018). Textural pattern classification for oral squamous cell carcinoma. J. Microsc..

[26] Rahman T.Y., Mahanta L.B., Das A.K., Sarma J.D. (2020). Automated oral squamous cell carcinoma identification using shape, texture and color features of whole image strips. Tissue Cell.

[27] Rahman A.U., Alqahtani A., Aldhaferi N., Nasir M.U., Khan M.F., Khan M.A., Mosavi A. (2022). Histopathologic oral cancer prediction using oral squamous cell carcinoma biopsy empowered with transfer learning. Sensors.

[28] Warin K., Limprasert W., Suebnukarn S., Jinaporntham S., Jantana P. (2021). Automatic classifcation and detection of oral cancer in photographic images using deep learning algorithms. J. Oral. Pathol. Med..

[29] Camalan S., Mahmood H., Binol H., Araújo A.L.D., Santos-Silva A.R., Vargas P.A., Lopes M.A., Khurram S.A., Gurcan M.N. (2021). Convolutional neural network-based clinical predictors of oral dysplasia: Class activation map analysis of deep learning results. Cancers.

[30] Musulin J., Štifanić D., Zulijani A., Ćabov T., Dekanić A., Car Z. (2021). An enhanced histopathology analysis: An AI-based system for multiclass grading of oral squamous cell carcinoma and segmenting of epithelial and stromal tissue. Cancers.

[31] Das M., Dash R., Mishra S.K. (2023). Automatic detection of oral squamous cell carcinoma from histopathological images of oral mucosa using deep convolutional neural network. Int. J. Environ. Res. Public Health.

[32] Lin H., Chen H., Weng L., Shao J., Lin J. (2021). Automatic detection of oral cancer in smartphone-based images using deep learning for early diagnosis. J. Biomed. Opt..

[33] Das N., Hussain E., Mahanta L.B. (2020). Automated classification of cells into multiple classes in epithelial tissue of oral squamous cell carcinoma using transfer learning and convolutional neural network. Neural Netw..

[34] Panigrahi S., Das J., Swarnkar T. (2022). Capsule network based analysis of histopathological images of oral squamous cell carcinoma. J. King Saud. Univ. Comput. Inf. Sci..

[35] Myriam H., Abdelhamid A.A., El-Kenawy E.S.M., Ibrahim A., Eid M.M., Jamjoom M.M., Khafaga D.S. (2023). Advanced meta-heuristic algorithm based on Particle Swarm and Al-biruni Earth Radius optimization methods for oral cancer detection. IEEE Access.

[36] Panneerselvam K., Nayudu P.P. (2023). Improved Golden Eagle Optimization Based CNN for Automatic Segmentation of Psoriasis Skin Images. Wirel. Pers. Commun..

[37] Erkan U., Toktas A., Ustun D. (2023). Hyperparameter optimization of deep CNN classifier for plant species identification using artificial bee colony algorithm. J. Ambient. Intell. Human. Comput..

[38] Vinaykumar V.N., Babu J.A., Frnda J. (2023). Optimal guidance whale optimization algorithm and hybrid deep learning networks for land use land cover classification. Eurasip J. Adv. Signal Process..

[39] Anilkumar Gona M., Subramoniam R. (2023). Swarnalatha, Transfer learning convolutional neural network with modified Lion optimization for multimodal biometric system. Comput. Electr. Eng..

[40] Subashchandrabose U., John R., Anbazhagu U.V., Venkatesan V.K., Thyluru Ramakrishna M. (2023). Ensemble Federated Learning Approach for Diagnostics of Multi-Order Lung Cancer. Diagnostics.

[41] Saab S., Saab K., Phoha S., Zhu M., Ray A. (2022). A multivariate adaptive gradient algorithm with reduced tuning efforts. Neural Netw..

[42] Wang S.-H., Phillips P., Sui Y., Liu B., Yang M., Cheng H. (2018). Classification of Alzheimer’s disease based on eight-layer convolutional neural network with leaky rectified linear unit and max pooling. J. Med. Syst..

[43] Szegedy C., Vanhoucke V., Ioffe S., Shlens J., Wojna Z. Rethinking the inception architecture for computer vision. Proceedings of the IEEE Conference on Computer Vision and Pattern Recognition.

[44] Howard A.G., Zhu M., Chen B., Kalenichenko D., Wang W., Weyand T., Andreetto M., Adam H. (2017). MobileNets: Efficient convolutional neural networks for mobile vision applications. arXiv.

[45] Tan M., Le Q.V. (2020). EfficientNet: Rethinking Model Scaling for Convolutional Neural Networks. arXiv.

[46] Abdollahzadeh B., Soleimanian Gharehchopogh F., Mirjalili S. (2021). Artificial gorilla troops optimizer: A new nature-inspired metaheuristic algorithm for global optimization problems. Int. J. Intell. Syst..

[47] Sayour M.H., Kozhaya S.E., Saab S.S. (2022). Autonomous robotic manipulation: Real-time, deep-learning approach for grasping of unknown objects. J. Robot..

[48] Saab S., Fu Y., Ray A., Hauser M. (2022). A dynamically stabilized recurrent neural network. Neural Process. Lett..

[49] Histopathologic Oral Cancer Detection Using CNNs. https://www.kaggle.com/ashenafifasilkebede/dataset?select=val.

[50] Rahman T.Y., Mahanta L.B., Das A.K., Sarma J.D. (2020). Histopathological imaging database for oral cancer analysis. Data Brief..

[51] Lian Z., Zeng Q., Wang W., Gadekallu T.R., Su C. (2022). Blockchain-Based Two-Stage Federated Learning with Non-IID Data in IoMT System. IEEE Trans. Comput. Soc. Syst..

[52] Aubreville M., Knipfer C., Oetter N., Jaremenko C., Rodner E., Denzler J., Bohr C., Neumann H., Stelzle F., Maier A. (2017). Automatic Classification of Cancerous Tissue in Laserendomicroscopy Images of the Oral Cavity using Deep Learning. Sci. Rep..

[53] Alkhadar H., Macluskey M., White S., Ellis I., Gardner A. (2021). Comparison of machine learning algorithms for the prediction of five-year survival in oral squamous cell carcinoma. J. Oral. Pathol. Med..

[54] Alhazmi A., Alhazmi Y., Makrami A., Salawi N., Masmali K., Patil S. (2021). Application of artificial intelligence and machine learning for prediction of oral cancer risk. J. Oral. Pathol. Med..

[55] Arikumar K.S., Deepak Kumar A., Gadekallu T.R., Prathiba S.B., Tamilarasi K. (2022). Real-Time 3D Object Detection and Classification in Autonomous Driving Environment Using 3D LiDAR and Camera Sensors. Electronics.

[56] Welikala R.A., Remagnino P., Lim J.H., Chan C.S., Rajendran S., Kallarakkal T.G., Zain R.B., Jayasinghe R.D., Rimal J., Kerr A.R. (2020). Automated Detection and Classification of Oral Lesions Using Deep Learning for Early Detection of Oral Cancer. IEEE Access.

[57] Shavlokhova V., Sandhu S., Flechtenmacher C., Koveshazi I., Neumeier F., Padrón-Laso V., Jonke Ž., Saravi B., Vollmer M., Vollmer A. (2021). Deep Learning on Oral Squamous Cell Carcinoma Ex Vivo Fluorescent Confocal Microscopy Data: A Feasibility Study. J. Clin. Med..

